# Cryptographic Considerations for Automation and SCADA Systems Using Trusted Platform Modules

**DOI:** 10.3390/s19194191

**Published:** 2019-09-27

**Authors:** Alexandra Tidrea, Adrian Korodi, Ioan Silea

**Affiliations:** Department of Automation and Applied Informatics, Faculty of Automation and Computers, University Politehnica Timisoara, 300223 Timisoara, Romania; alexandra.tidrea@gmail.com (A.T.); ioan.silea@upt.ro (I.S.)

**Keywords:** security, SCADA, automation systems, TPM, Modbus TCP, HMAC, ECDSA, cryptography, industrial internet of things

## Abstract

The increased number of cyber threats against the Supervisory Control and Data Acquisition (SCADA) and automation systems in the Industrial-Internet-of-Things (IIoT) and Industry 4.0 era has raised concerns in respect to the importance of securing critical infrastructures and manufacturing plants. The evolution towards interconnection and interoperability has expanded the vulnerabilities of these systems, especially in the context of the widely spread legacy standard protocols, by exposing the data to the outside network. After gaining access to the system data by launching a variety of attacks, an intruder can cause severe damage to the industrial process in place. Hence, this paper attempts to respond to the security issue caused by legacy structures using insecure communication protocols (e.g., Modbus TCP, DNP3, S7), presenting a different perspective focused on the capabilities of a trusted platform module (TPM). Furthermore, the intent is to assure the authenticity of the data transmitted between two entities on the same (horizontal interoperation) or different (vertical interoperation) hierarchical levels communicating through Modbus TCP protocol based on functionalities obtained by integrating trusted platform modules. From the experimental results perspective, the paper aims to show the advantages of integrating TPMs in automation/SCADA systems in terms of security. Two methods are proposed in order to assure the authenticity of the messages which are transmitted, respectively the study presents the measurements related to the increased time latency introduced due to the proposed concept.

## 1. Introduction

Automation and SCADA (Supervisory Control and Data Acquisition) systems are in charge of controlling and monitoring plants and critical infrastructures, vital for the well-functioning of important areas such as water, food, health, economy, transportation, energy, and national security. In the current context of a more and more digitalized physical world [1,2], it is more than necessary to protect and secure these systems against any type of attack. The integration of the physical industrial process in the virtual world is the first step of Industry 4.0, which is being considered as the fourth industrial revolution due to generation of opportunities regarding economic growth in the context of sustainability and the enabling of mechanism which are allowing the real-time communication between machines and between machines and manufactures [3]. The enabling of the mechanisms responsible for interconnection, self-monitoring, and self-control is done though digital platforms. Several challenges of digital platforms within Industry 4.0 implementation, in addition to the high costs of updating the legacy systems, are related to the lack of trust between providers and suppliers since sensitive information is involved, to data security and to data ownership [4]. Starting with the need for interoperability and interconnectivity in the context of Industrial-Internet-Of-Things (IIoT) and cyber-physical systems (CPS), an air-gap network is no longer considered as efficient in securing the infrastructures [5]. Concerns regarding the protection and necessity of research for providing viable security solutions were raised in the last few years.

Applying IIoT and Industry 4.0 concepts in the industry, the interoperability of the entities has to be assured both vertically [6] and horizontally [7] regarding the hierarchical levels, meaning that sensing devices and actuators from the field level, as well as Programmable Logic Controllers (PLCs), microcontrollers, microcomputers from the control level, SCADA systems, historians from the fog or cloud, have to vehiculate data on the same level (e.g., PLC to PLC) or on different levels (e.g., frequency converter to PLC, PLC to SCADA, sensing device to historian). 

The industry contains chronologically and technologically dispersed intelligent devices due to their long lifecycle. The so-called legacy systems are therefore very present assuring the heterogeneity of the existing protocols [8,9]. Despite the lack of security due to legacy structures, legacy automation and SCADA systems are widely used. The industry does not want to replace existing functioning solutions and therefore interoperation and other IIoT concepts are applied as non-invasively as possible. One approach presented in [10] is to adopt a smooth migration from the legacy manufacturing systems towards the Industry 4.0 concept by offering an Ethernet-based communication network in the context of SCADA and automation systems. Another approach is presented in [11] where the authors are implementing a program which reconfigures machine parameters and performs self-data analysis in order to automatize a bottling process. The issues are to obtain interoperability and security. Interoperability is usually obtained through gateways/wrappers targeting the basic protocols (e.g., the largest application legacy protocol Modbus [12], and others like IEC 61850 [13], 61499 [14], etc.) towards OPC Unified Architecture (OPC UA). The security is obtained usually by OPC UA [15,16], or other gateway-based strategies [17]. However, in many cases the basic protocol (usually Modbus TCP) is not subject to sudden change being already functioning in practice to vehiculate data between PLCs or other types of control devices, between frequency converters or sensing devices and control devices, and in the control device to SCADA integration either directly into the SCADA software or into centralizing PC based OPC/OPC UA servers (e.g., KepServerEx). 

In the above-mentioned context, the authors propose an approach for legacy protocols from industrial systems to secure the vehiculated data based on trusted platform modules (TPMs). The proof of concept is emphasized for the widely spread Modbus TCP legacy protocol and the entire research is developed and assessed in a close relation to practice, in order to gain higher applicability and technological readiness level. 

The paper describes, in Section 2, the relevant literature studies for the subject of this paper. The purpose of Section 3 is to demonstrate the replication of a successful MITM (Man-In-The-Middle) attack based on studies done in respect to the practical attacks against automation and SCADA systems. Section 4 contains the description of the proposed concept from experimental setup to design towards implementation. Section 5 contains the experimental results, their interpretation and the discussions related to the obtained results. In Section 6, the research is concluded.

## 2. Related Work

The literature addresses the security issues related to the critical infrastructure by covering different levels of the architecture. From the protocol communication point of view in [18] it is stated that the Modbus is the most frequently used protocol in SCADA and automation systems, even though it was rated as being insecure [19]. As described in [20], one important aspect to be considered in the analysis of automation and SCADA structures is related to the PLC (programmable logic controller) security in respect to payload and firmware attacks.

In this context, the attacker will try to gain access through compromising software applications in order to install an access point. Changing the execution mode of the automation process can cause operational disturbances or physical damage. The most known major attack against industrial control systems is called “Stuxnet”, built to interrupt Iran’s nuclear program in an isolated system. According to [21], the virus has exploited Windows operating system vulnerabilities, Siemens PCS 7 industrial software applications, WinCC, STEP 7, and Siemens PLCs. The communication between WinCC software and Siemens PLCs was intercepted via the s7otxbxdx.dll library that made it possible to uninstall PLCs and impersonate a normal PLC behavior as shown by [22]. The discovery of the virus in 2010 raised awareness in respect to security issues in industrial control systems.

### 2.1. Vulnerabilities of SCADA and Automation Systems

In 2018, the study done by Kaspersky Lab [23] on computers from industrial control systems, using Kaspersky Security Network, showed an increase of 4.6% over the year 2017 in terms of number of attacks on industrial systems. Multiple SCADA software vendors are offering the possibility to monitor industrial processes through mobile devices, which raises new issues, as shown in the analysis done by Embed Security [24] which identified many security vulnerabilities such as insecure authentication, insecure communication, inefficient cryptography (hard-coded encryption keys, cryptographic schemes applied in an incorrect manner), lack of methods to prevent code manipulation. Attackers can thus compromise communication channels by sending artificial data to mobile applications with the intention of infecting devices and accessing the SCADA infrastructure layers.

SCADA security systems are vulnerable to attacks on hardware, software, and communication protocols. In [25], as major vulnerabilities for SCADA automation systems, the following were identified: “zero-day” vulnerabilities, lack of task prioritization, database injection, and issues at communication protocol level. Additionally, in [26], the vulnerabilities are highlighted at the level of interaction with HMI (Human-Machine Interface): cross-site scripting, the existence of unused ports and services, vulnerabilities which are allowing unauthorized writings leading to buffer overflow. In [27] the authors emphasize the lack of authenticity, lack of integrity, and lack of confidentiality for the classical network communication protocols used in the automation and SCADA systems which are allowing intruders to perform denial-of-service, man-in-the-middle, message spoofing, replay or data injection attacks. New communication protocols were developed in order to include a security mechanism as it is stated in [28]. Nevertheless, it is a big challenge to replace the classical protocols with the new ones due to the high number of interconnected systems. Therefore, a method for introducing security mechanisms is required.

### 2.2. Security Solutions for Industry 4.0

In order to respond to the vulnerabilities identified for the SCADA and automation systems, various methods were proposed in literature. The authors will mention the relevant ones in respect to the proposed concept of this paper, which addresses the issues related to the communication protocols and hardware trusted module integration. Lack of a method for master-slave authentication is one of the problems identified in the Modbus protocol. In order to respond to this problem, in [29] the authors proposed a one-way authentication method based on a hash function, which involves authenticating the commands received from the master by the slave device. Scheme authentication assumes a request from master to slave in order to execute a single command from one vector of n possible commands and generation of a hash chain for each command and has the purpose to prevent master impersonation which could send unwanted messages to the slave. In 2012, in [30] the inadequacy of the proposed solution from [29] is demonstrated by launching basic attacks on the authentication protocol, which reveals that there may be cases where the slave can no longer authenticate or instances where unwanted orders are executed by the master. Following a similar direction, in [31] the authors presented a new method based on stream transmission control protocol (SCTP) and authentication messages based on keyed-hash functions (HMAC) in order to provide a security solution for transactions on Modbus TCP and to establish a mutual authentication mechanism. In this case, all the solutions are software-based and do not include an additional hardware for securing the storage of the secret keys used.

Another approach is presented in [32] where an intrusion detection system is proposed on the clustering of the sequences corresponding to the attacks collected through honeypot for Modbus TCP protocol called Conpot. Tests which were conducted for over a month on a smart meter with Modbus communication have proven a high accuracy in identifying attack patterns. For addressing the lack of an effective integrity method, in [33] the authors proposed a solution for improving security through the covert channel concept. It offers the possibility of creating a communication channel outside the normal communication paths, which implies a certain degree of integrity verification between devices. However, the method raises problems regarding the introduction of delays on the communication channel which could lead to operational delays. A secure version of the Modbus TCP protocol was published by introducing TLS as packet encapsulation method sent to Modbus to provide authenticity and integrity [34]. The new protocol will use a new port unlike the original version of the protocol, namely port 802. However, a detailed analysis of the latency introduced by the new secured protocol has not been made. An eloquent example of overhead and increased latency has the solution proposed by [35] that aims to secure the Modbus RTU communication protocol with the Shamir method, with the cost of introducing an increased overhead as is stated in [36].

Another subject for research in relation to the IIoT concept is the possibility of introducing a new security mechanism in the critical infrastructure architecture [37,38], in order to protect the SCADA and automation systems against cyber-attacks and satisfy the need for enhanced security. In this case, only software-based solutions are proposed. Regarding the use of digital signatures as a security mechanism, in [39], there are elliptic curves with different key sizes analyzed, having the performance part of the software implementation of Elliptic Curve Digital Signature Algorithm (ECDSA) evaluated by testing it on a wireless sensor network. Furthermore, in [40], a concept for securing the smart building automation using an Infineon OPTIGA TPM as hardware security module is proposed, which includes TLS and the utilization of the ECDSA algorithm for the gateway authentication process, but no measurement related to the execution of the cryptographic functions is presented.

In the literature, several hardware-based security solutions were proposed along with the software security solution, such as in [40], where the TPM is used in order to create a trusted chain for the IoT devices which are part of an IoT Cloud infrastructure. Nevertheless, in this case, only a theoretical approach is illustrated. In [41] a method for authentication of smart sensor with a router which has associated a TPM is presented, in order to provide a solution for machine-to-machine communication in an IoT Environment. Literature is also approaching the enhancement of the SCADA and automation system by using the TPM in order to ensure hardware security along with the software security, an action necessary due to the evolution of the systems towards Industry 4.0 [42,43]. 

## 3. SCADA and Local Automation System Setup: Man-In-The-Middle Attack on Modbus TCP Protocol

The Modbus TCP messages are transmitted in plain text without any security mechanisms applied. Therefore, an attacker, once gained access to the network, will be able to impersonate the master or slave through different techniques as described in [44] and to send modified messages in order to disrupt the normal operation of an automation process.

**Figure 1:**
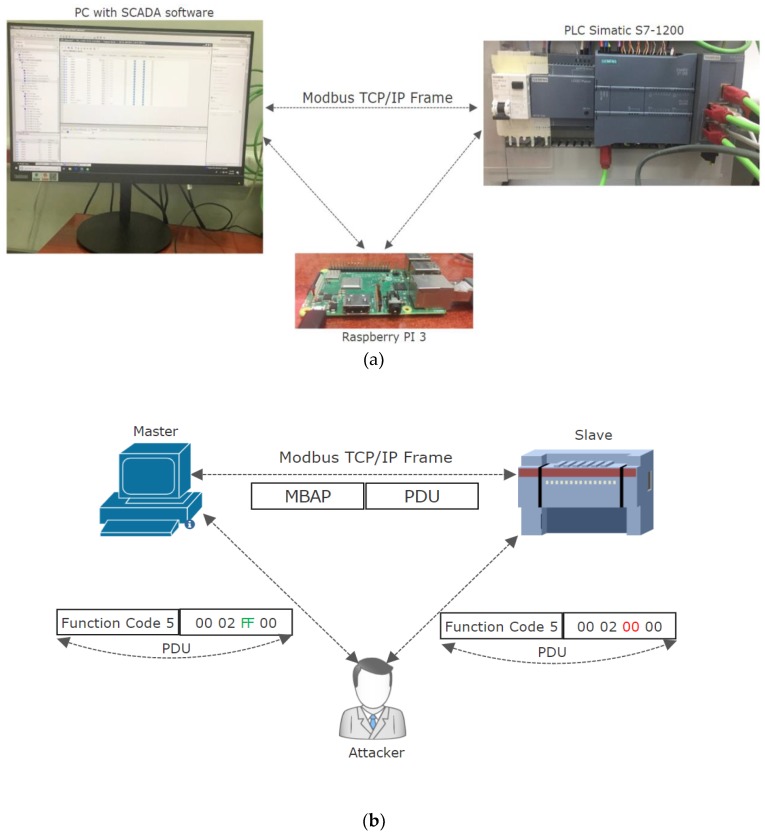
MITM (Man-In-The-Middle) physical setup and results of the attack. (**a**) Real setup for deploying the attack. (**b**) MITM resulted in Modbus TCP request message altering.

In order to replicate a MITM (Man-In-The-Middle) attack on a setup with a real PLC, we have deployed the MITM on a SCADA and local automation system which had a PC acting as a master station and a Siemens S7-1200 PLC acting as a slave connected through an Ethernet switch. The description of the real setup used is shown in Figure 1a. The attacker station was connected to the same Ethernet switch, being designed to have the following components:

- Kali Linux as operating system since it is designed for allowing the integration of a variety of tools used for penetration testing and hacking.
- Ettercap tool for performing the MITM attack since it allows Address Resolution Protocol (ARP) sniffing, ARP poisoning and it allows to design filters for manipulation of Modbus TCP protocol [45].
- T-shark for communication monitoring in order to analyze the Modbus TCP traffic [46].

Using the Ettercap tool, the malicious station was able to employ the option of ARP poisoning in order to impersonate the master and to modify the messages sent over Modbus TCP.

The structure of a Modbus TCP message frame is composed of Modbus Application Protocol Header (MBAP) and Protocol Data Unit (PDU). The PDU contains the function code and additional information in respect to the data requested by the master station or the function code and the data read as a response sent by the slave. As described in Figure 1b, by using an Ettercap filter, the request from the master station for setting on a coil was intercepted by the attacker, modified in order to request the setting of the coil to OFF, and sent to the PLC. Since the malicious station was acting as the original master, the PLC executed the request by setting the coil to the requested value.

The Function code 5 represented by the value *05* is used for requesting the setting of a coil, the first two bytes from the data are describing the address of the register where the data should be written, followed by the value to be written where *FF* is associated with the ON value and *00* with the OFF value.

Using the same design as the one defined for this scenario, a malicious station was built and used for testing the proposed concept, in order to evaluate the protection against MITM attacks.

## 4. Proposed Concept

In the context of IoT, the horizontal/vertical interoperability of SCADA/automation systems represents a challenge in respect to security and protection, since the tendency is to expose the manipulation, control, and monitoring of data associated to industrial processes remotely. 

In what concerns the horizontal interoperability between the components of local automation systems, the devices such as PLCs are transferring data between each other through Modbus, if one of the devices is configured as the master. On the other hand, for SCADA applications the interoperability is realized mostly vertically by exchanging information with the devices or sensors through various protocols such as Modbus over TCP. Further, driven by the evolution of the systems of interest, the integration of vertical applications in order to fulfill different tasks in the context of smart factories or intelligent buildings, is creating the need for assuring horizontal interoperability at a higher level. 

For a SCADA application, the Modbus TCP data can be exposed through an OPC UA server, which can be configured among other possibilities to send the Modbus data messages embedded in the OPC UA transport. For the automation systems, the Modbus TCP is exposed trough code which can be designed, configured, and downloaded to the devices placed at the lower level of system architecture (PLCs). Worth mentioning is that Modbus TCP is lacking security mechanisms in contrast with the necessity for protection against cyber attacks imposed by the need for increased and complex interoperability beyond the limits of a closed system. Additionally, considering the fact that Modbus TCP is used for assuring both horizontal/vertical communication between the SCADA and local automation systems component, which makes it a placeholder for other communication protocols, the authors selected it for implementing the proposed concept on a real SCADA and automation system.

Considering the structure of SCADA and automation systems, the author’s approach has the goal to present a solution for securing the network communication between a master and a slave device in the context of SCADA and automation systems architecture, by using a hardware trusted platform. Moreover, the intent of the paper is to prove through experiments the integration and applicability of the hardware trusted platform module in the context of systems of interest. The proposed concept will emphasize the usability of the TPM in securing the network communication and will evaluate the latency introduced by each of the applied cryptographic operations.

**Figure 2:**
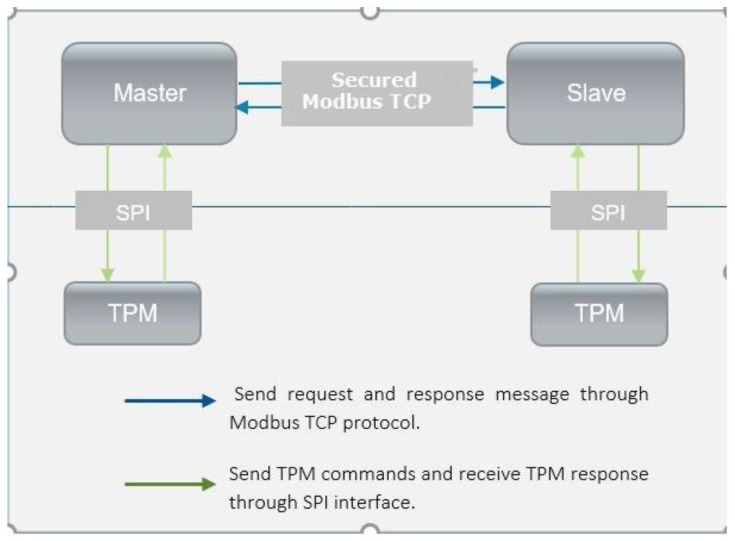
System architecture abstract view.

From architecture perspective described in Figure 2, the concept is to have two independent system elements, a master and a slave, that are communicating through the Modbus TCP protocol which was chosen for demonstration purposes and for lack of security. 

Each system element has associated a TPM 2.0, which is intended to be used for secured key storage, assuring the hardware security and also as a crypto microprocessor. The TPM specification were designed by the Trusted Computing Group (TGC) [47] available as ISO/IEC 11889 in order to offer a hardware-based security solution by enabling features such as enhanced authorization, quick key loading, generation and secure storage of keys, encryption and decryption mechanisms for both keys and data. It has to be pointed out that the main features offered by the TPM and used in this paper are secure key storage and a mechanism for executing cryptographic functions.

The proposed architecture must be considered as a proof-of-concept since it is not deployed yet on a real automation/SCADA infrastructure, but it is tested on an experimental setup having two devices which emulates the transmission of data acting as one.

### 4.1. Secure Key Storage with TPM

The TPM’s approach regarding key management involves a hierarchy system based on different security roles. More than that, each key has an individual security protocol, which can be among other options either a password or an enhanced authorization policy. Due to non-volatile memory restrictions, not all the keys are stored in the persistent memory, but the TPM has the option to generate them or to load external keys. The loading of the keys stored in the TPM is done by using the key cache mechanism, which has the purpose to offer fast access, by being encrypted by using the symmetric key mechanism. The following functions were used to create and store keys either in the context or in the persistent memory.

- TPM2_CreatePrimary which allows the creation of a key with an associated seed stored in the persistent memory.
- TPM2_Create is used to create an encrypted private and public key which, with the correct settings, can be associated in the hierarchy under the primary key. In these cases, the keys will not be saved in the persistent memory, since they will be loaded from the TMP cache or recreated.
- TPM2_Load and TPM2_LoadExternal will be used to load the keys.
- TPM2_NVWrite is used for writing and protecting the keys considered to be critical for the proposed concept.

### 4.2. Cryptographic Authentication Scheme

The author’s proposed concept has as the main actor the TPM and it analyzes the usability of it in what concerns the protection of the system of interest against security attacks. 

Based on the capabilities of TPM, two methods are proposed and evaluated on the experimental setup as the proof of assuring the authenticity of the data transmitted between a master and slave device, by highlighting the advantages of a secure cryptographic microprocessor. 

In order to set up the key hierarchy and to assure trust and secure key storage, a trusted root key is created with the TPM and stored encrypted in the non-volatile memory (NVM). The signing key which will be created or loaded in the TPM will be associated with the root key, which will protect against key tampering from outside of the TPM. In order to be able to benefit the TPM capabilities and to implement the proposed concept, an endorsement key, based on an RSA template which uses RSA-2048, AES-128, and SHA-256 was created once for each of the trusted platform modules. Based on the endorsement key, an ECDSA-256 primary key was created and resulted in a key pair, which contains a public key accessible externally from the TPM and a private key stored in the TPM.

The first method responds to the need of protecting the data in case of alteration and injection of a new message between a master and a slave device by using the HMAC-SHA256 algorithm. In case an adversary intercepts the message, the resulted keyed-hash cannot be re-computed without knowing the cryptographic key. Therefore, one of the major concerns for the algorithm consists of defining efficient methods for protecting it. In this case, the TPM will bring as an advantage a secure storage of the key, but also an additional cost compared to a solution without additional cryptographic hardware, in which the key used in HMAC algorithm will be processed and stored by the software solution itself.

Due to TPM specification, the HMAC key cannot be extracted from the TPM after being created through TPM and stored in the NVM secure memory. Nevertheless, in order to be able to use the capability of the TPM regarding the secure key storage, it is necessary that the sequence below is performed once, in a secure environment, by a trusted third party, when both master and slave are connected with the associated TPMs for the first time:

- Generate externally from the TPM a HMAC key *k* of 256 bit which will be used for all the devices which will have an associated TPM, where *k* is considered a symmetric key used for TPM keyed hashing.
- Generate through each TPM associated an RSA key pair (*kPriv, kPub*) for each of the devices intended to be used, where *kPriv* is the private key stored securely in the TPM persistent memory and the *kPub* is stored in the public key in the non-secure memory.
- Export *kPub* and load it into the non-secure memory of each device.
- Encrypt the HMAC key *k* with the RSA *kPub* through the TPM and store it encrypted in the non-secure memory of each device. Afterwards, when the system is running, an adversary will not be able to decrypt the HMAC key without the RSA *kPriv* stored securely in the TPM memory. Worth mentioning is that each device will have a different RSA key pair (*kPriv, kPub*) since it is created by each TPM connected to each device. Furthermore, the proposed concept for the authentication scheme is described as follows:
- At system startup both master and slave will perform:
  1. Decrypt the HMAC key using the TPM with the RSA *kPriv*.
  2. Load the decrypted HMAK key into the TPM and remove it from volatile memory of the device.
- At system runtime the communication between master and slave will be performed:
  1. *ComputeHMAC (k, m)* where the master device computes a keyed hash with *k* as secret key of the message *m* obtaining a keyed-hash hash *h*.
  2. *ConcatenateHash (m, h)* where the master device adds the hashed message *h* to the message *m* and send it to the slave device.
  3. *CheckHash (m, h)* where the slave device computes a keyed-hash with *k* as secret key of the message *m* and compares it with what it received from the master device.
  4. *AcceptRequest* where the slave device accepts the request received from the master device because the keyed hash values were the same.
  5. *AbortCommunication* where the slave device does not execute the command requested by the master device because the keyed hash values were different.

The procedure for HMAC computation *ComputeHMAC (k, m)* using TPM available functions is as follows:

(a) *StartHMAC* sequence using SHA-256 as algorithm for transmitted message *m* digest.
(b) *UpdateHMAC* sequence with the Modbus TCP *m* in order to be hashed with the HMAC key *k.*
(c) *CompleteHMAC* sequence by returning the hashed message *h*, which will have 256 bit length.

The same protocol is applied for the response sent by the slave device towards the master, assuring that the data was not tampered and the message is authentic. Nevertheless, the proposed approach does not protect against replay attacks. In order to respond to this need, further enhancement is needed by adding either a nonce or a timestamp for each message. Worth mentioning is that the nonce can be generated by using the random number generator provided by the TPM, which is a true random generated number implemented through hardware. In this case the HMAC-SHA256 will be performed as follows:

- *ComputeHMAC (k, (nonce || m))* where the device computes a keyed hash with *k* as secret key of the message *m* concatenated with a randomly generated *nonce* or
- *ComputeHMAC (k, (timestamp || m))* where the device computes a keyed hash with *k* as secret key of the message *m* concatenated with the message *timestamp.*

The second method proposed has the goal to demonstrate the applicability of Elliptic Curve Digital Signature Algorithm (ECDSA) in assuring the authenticity of the transmitted data between a master and a slave device. The ECDSA uses Elliptic Curve Cryptography (ECC) to produce signatures based on the private key from a key pair (*kPrivECDSA, kPubECDSA*). In this case *kPrivECDSA* is an integer corresponding to the private key and *kPubECDSA* is an elliptic curve point corresponding to the public key. The method will be supported by the trusted hardware platform TPM 2.0, in order to respond to the security concerns related to the secure key storage of the private keys. In this case, an attacker will not be able to produce a valid signature over a modified message without knowing the private key of the entity responsible for issuing the digital signature. In order to check the validity of computed digital signature, both master and slave must know the *kPubECDSA* of the other one. Therefore, it is necessary to perform the following steps in a secure environment for the first time when the devices are connected with the associated TPMs:

- Generate the key pair *(kPrivECDSA, kPubECDSA)* by the TPM using ECC-256 for each device.
- Store the *kPrivECDSA* in the persistent memory of the TPM and *kPubECDSA* in the context of the TPM.
- Load externally the *kPubECDSA* of the master device into the TPM of the slave device.
- Load externally the *kPubECDSA* of the slave device into the TPM of the master device.

The wolfTPM offers a possibility to select several curves for the generation of the ECC key pairs, among which NIST-P192, NIST-P224, NIST-P-256, NIST-P384, and NIST-521. For the generation of the key pairs, we selected the SHA-256 algorithm and the curve NIST-P-256, since they are supported by the Infineon OPTIGA TPM module. The steps regarding the cryptographic scheme are described in detail for the master acting as an initiator of the authentication and the slave acting as a verifier of the computed signature:

- At system startup the following actions will be performed:
  1. Load the *kPubECDSA* of the slave device into the TPM of the master device from the master device non-secure persistent memory.
  2. Load the *kPubECDSA* of the master device into the TPM of the slave device from the slave device non-secure persistent memory.
- At system runtime the communication between master and slave will be performed:
  1. *SignMessage (kPrivECDSA, m)*, where the master device computes a digital signature with *kPrivECDSA* as a secret key of the message *m,* obtaining a signature *S* of 512 bit length.
  2. *ConcatenateSignature (m, S*) where the master device adds the signature *S* to the message *m* and sends it to the slave device.
  3. *VerifySignature (kPubECDSA, S*) where the slave device computes a verification of the received signature using the master *kPubECDSA* and compares it with the expected response.
  4. *AcceptRequest* where the slave device accepts the request received from the master device because the signature is valid.
  5. *AbortCommunication* where the slave device ends the communication with the master device because the signature is not valid.

The protocol is applicable for bidirectional communication between the master and slave.

### 4.3. Experimental Setup

**Figure 3:**
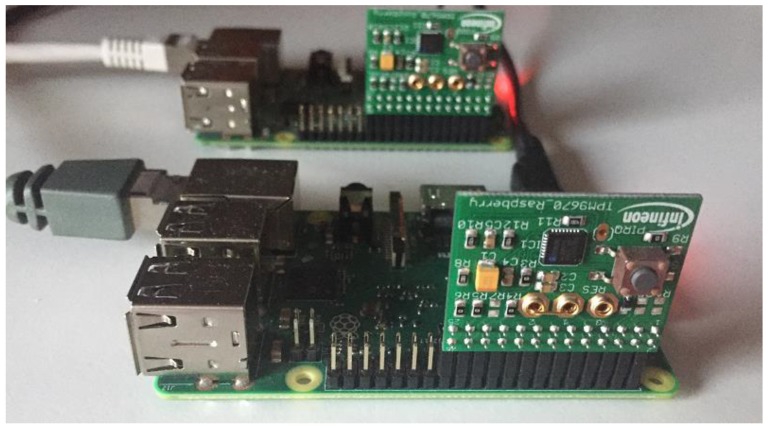
Experimental setup with two Raspberry Pi boards and two Infineon TPM2.0 modules.

In order to emulate a Master node which communicates with a slave node through Modbus TCP, we chose a Raspberry Pi 3 for each node due to very few options in respect to open-source available software for master-slave node emulation in the context of SCADA and automation systems architecture. For implementing the cryptography functions and key storage, an OPTIGA TPM SLB9670 TPM2.0 from Infineon was connected through Serial Peripheral Interface (SPI) interface to each of the Raspberry PI 3. The TPM was chosen since it brings, in addition to hardware security, an environment for cryptographic computation, which has the advantage of reducing the time for an application which without the TPM would be making all the computations and will be storing all the keys in a non-secure environment. The experimental setup from the hardware perspective is shown in Figure 3.

From the software perspective, several open source C language libraries were used in order to achieve the proposed functionality for the hardware setup, as follows:

- Libmodbus for Modbus TCP protocol [48].
- Mbpoll for master Modbus polling and message request [49].
- wolfTPM library for application integration [50,51].

The Libmodbus was chosen due to its scalability and its design, which follows the Modbus specification and implementation guide. More than that, it has the capability to send/receive messages acting both as master or as slave. For the Master node, this library was integrated with the Mbpoll application which is used for sending requests towards the slave node. For emulating the communication on the slave node, the Libmodbus was updated by adding a functional application layer in order to listen for requests and to integrate the cryptographic functions.

In order to use the TPM 2.0 specification, the Linux kernel for the Raspberry needed to be updated and built in order to enable the TPM 2.0 driver. Regarding the enabling of communication with the TPM2, the TPM2 2.0 software stack [52] was used together with the wolfTPM library. The TPM 2.0 software stack is providing Enhanced System API (ESAPI), TPM Command Transmission Interface (TCTI), Resource Manager (RM) support for cryptographic functions, random number generation, remote attestation, key secure storage, and secure boot capabilities. The TPM 2.0 software stack was used for TPM integration testing only, in order to check if the hardware module is accessible through commands and to evaluate if the cryptographic functions are executed as expected. In order to send commands to the TPM over the SPI interface of the Raspberry PI by using ESAPI and to integrate them in the Libmodbus software, the wolfTPM was selected since it is providing well-structured wrapping functions available as software modules over the native TPM commands. 

**Figure 4:**
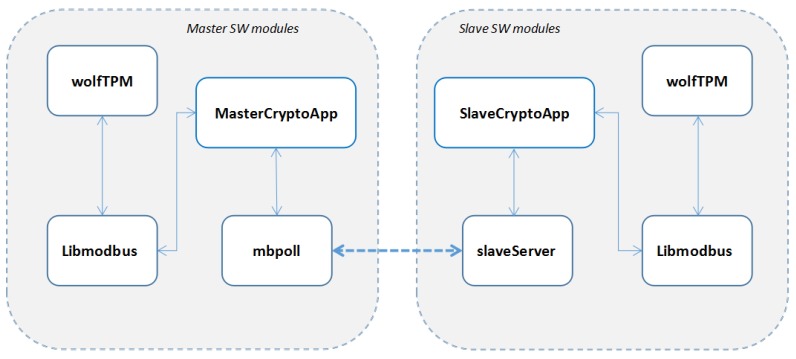
Software application modules.

The application implemented and deployed on the experimental setup is divided into several modules as defined in Figure 4. Each of the software modules is fulfilling well defined tasks and it is designed to have an efficient communication with the other modules from number of interfaces perspective.

The wolfTPM software modules responsible for the APIs related to the secure key handling and to the cryptographic commands chosen for the proposed concept were integrated with the Libmodbus modified software. In order to apply the cryptographic functions over the Modbus TCP communication, two functional application layers MasterCryptoApp and SlaveCryptoApp were implemented over Libmodbus. 

## 5. Experimental Results and Discussion

To evaluate the proposed concept based on TPM functionalities, the duration of each function executed by the TPM part of the cryptographic authentication scheme was measured. Furthermore, the latencies introduced by the cryptographic functions were analyzed in contrast with the initial Modbus TCP functions. Another target in the respect of the results is represented by the strength of the security level introduced by the proposed concept. Since for the experimental setup two Raspberry Pi were used with Linux distribution, the time measurements were done using available software functions.

Since for each of the TPMs it is necessary to setup the key hierarchy and to create a trusted primary key, the paper presents the time measurements associated to the necessary cryptographic operations for key generation based on RSA in Table 1:

In this case the generation of the primary key is 0.040540 s higher than the retrieving of the RSA endorsement key template, since the execution of the cryptographic algorithms specified by the template is done once the generation of the key is requested.

In order to clearly distinguish between the results of the proposed concept in terms of duration for each cryptographic operation and the duration associated to the required operations done by third party, in Table 2, the computations of each cryptographic function done for the second case are illustrated. These results are presented to get a better perspective on the time of execution for the cryptographic functions using a TPM.

It can be observed that for the encryption of the HMAC key using the RSA algorithm, the total duration is 0.105162 s, where the most time-expensive cryptographic function is generation of the RSA key pair consisting of a public and a private part. Worth mentioning is that the total duration for the generation of the key pair and the loading of the public key, cryptographic operations associated to the ECDSA ECC-256 method is 0.023065 s higher than the time measured for the HMAC SHA-256 method. Due to the fact that these steps are done once in the life cycle of the system of interest, it has no impact during the normal functional operation. 

For the first cryptographic authentication scheme, the time for executing the functions was monitored and measured during a master-slave communication considering the experimental setup, where the master issues a request and the slave sends a response. The results described in Table 3 present the computed time during normal operation.

In Table 4, the measurements corresponding to the cryptographic scheme based on the ECDSA algorithm emphasize that the duration of the cryptographic operations are higher by 0.153731 s than the ones related to the HMAC algorithm, as expected. It can be observed that the duration of the cryptographic operations associated to the ECDSA algorithm executed at system runtime is 0.171494 s and the duration for the HMAC algorithm computation is 0.017763 s as presented in Table 3. Nevertheless, at startup, since it is necessary to decrypt the HMAC key using the RSA private key stored in TPM, the duration for performing the steps corresponding to the first method proposed will be 0.068935 s higher than for performing the ECDSA cryptography operations.

Aspects related to the algorithm’s strength selected for the concept and the size of their results reflected in additional bytes to be sent over Modbus TCP, are described in Table 5. Worth mentioning is that the strength of the proposed algorithm is given by the security strength of the key used. As mentioned in the security standard [53], for a HMAC-SHA256 the strength is 256 bit as the key size and for ECDSA ECC-256 the security strength is 128 bit, being considered as half of the key size. 

**Figure 5:**
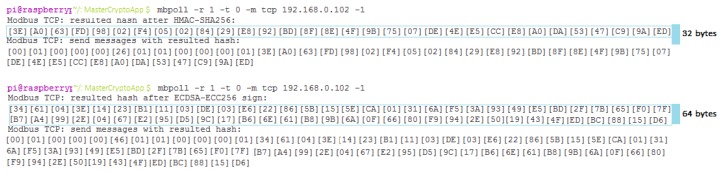
HMAC-SHA256 and ECDSA ECC-256 additional size introduced for the Modbus TCP message.

One of the advantages of the HMAC-SHA256 based method is that the resulted hash will introduce a 256 bit additional size in respect to the constructed Modbus TCP message compared to 512 bit size added by the ECDSA ECC-256 as is shown in Figure 5. The additional size will lead to an additional time in the communication network for sending or receiving messages over Modbus TCP.

**Figure 6:**
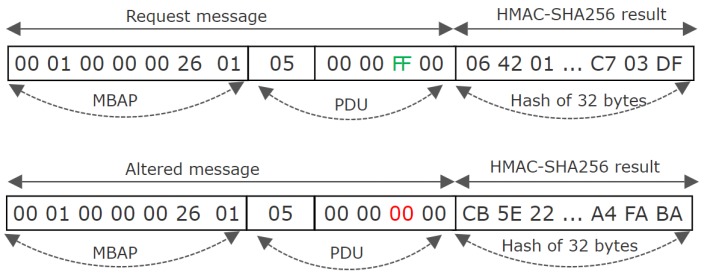
Modbus TCP message structure based on HMAC-SHA256 proposed concept.

A MITM attack was performed when using each of the proposed methods by connecting a malicious station to the same Ethernet switch used for the master and slave devices. For both methods the result was unsuccessful, but in order to offer clarity to the reader, the results are described in detail for the HMAC-SHA256 based solution. In this scenario associated to the HMAC-SHA256 proposed concept, the attacker intercepted the message, changed the data related to writing a coil, and sent the altered message to the slave. Once the request was received, the slave computed a keyed hash with the known secret key over the received message. Since the altered message had a different content than the original one, the resulted hash was different than the one received in the request, which lead to non-execution of the request and to the communication being aborted. In Figure 6 the request message and its additional computed keyed hashed is shown, respective the altered messaged and its additional computed keyed hashed. It can be observed that the obtained hashes are different and in this case the request will not be executed by the slave.

Furthermore, an attacker cannot reproduce a valid keyed-hash over a message without knowing the secret key, which in this case is protected by the TPM and it cannot be retrieved.

Considering the obtained measurements, we can state that the HMAC based solution is more efficient during runtime from the duration perspective than ECDSA proposed scheme. On the other hand, it can be argued that the ECDSA concept has the advantage of having the key pair generated and stored safely in the TPM, which makes it the proposed mechanism from this perspective, even if the key strength for the HMAC key is higher than the ECC-256 key strength. The obtained results are sustaining the realization of the aimed goals set for this paper, since two mechanisms for introducing security on the communication between two nodes of a network focused on TPM capabilities, were presented, implemented, and evaluated. Moving forward, the proposed concept might be adjusted for the same purposes as presented in this paper with applicability for other legacy communication protocols used in SCADA and automation systems or in a modern IoT architecture.

Similar to the TPM that we used in our experimental setup there are TPMs which are available on the market for industrial security such as OPTIGA™ TPM SLM 9670 TPM2.0 [54], which is qualified for industrial application with the purpose to be integrated as part of the system’s structure together with different devices such as PLCs, industrial PCs, or gateways. There are automation system components with SPI interface and open-source software such as the Controllino PLC controllers distributed by Vistion [55] which makes the OPTIGA TPM as a plug-and-play security device in the context of secure communication, secure key storage, and device authentication. In a scenario where we consider having PLCs with SPI interface and C code deployment, since the Modbus TCP is exposed through software, we will be able to implement the proposed concept. 

Furthermore, addressing the subject of deploying the proposed concept on a real SCADA and automation systems from a more general perspective, one must consider hardware and software changes. From hardware perspective it should be considered from the design phase to include additional interfaces for the devices in order to communicate with the TPMs or even embedding the TPM microchip as part of the hardware device. A few IoT gateways [56], responsible for saving data into cloud, which are already on the market, have added as part of their hardware design a TPM microchip further used for assuring the root of trust between the gateway and the cloud service provider. However, such gateways are not covering the security issues related to communication between devices inside the network, neither do they assure the authentication of the devices within the network. Except the changes of the existing hardware, software changes are required in order to communicate with the TPM and to use the cryptographic functions for introducing security from the beginning of the product lifecycle. Having the security in mind and considering the nowadays real cyber threats, one way for implementing these changes will be directly dependent on the vendors approach for designing the hardware and developing the core software. Another approach will be for one to develop smart gateways in order to cover the security needs for devices placed also at lower levels of a system.

## 6. Conclusions

This paper presents a TPM based working solution for both emphasizing the advantages of using a hardware trusted platform and for securing with it the communication between two entities of an automation/SCADA structure against an MITM attack. The work reproduced an MITM attack between a real SCADA system and local automation system in order to gain a better perspective of the existing vulnerabilities and to find a way to protect the nodes and the communication through security mechanisms. The design of the MITM attack was deployed afterwards on the experimental setup in order to assess the protection added by the proposed methods. One of the main advantages of TPMs, the capability of safely storing and generating a cryptographic key, is approached in the proposed concept for increasing the level of security, since the retrieving of data stored securely in the trusted hardware is not possible. This makes a strong case for using the TPMs as part of the methods developed for securing the critical infrastructure. Furthermore, all the cryptographic operations described in the authentication scheme are executed by the TPM itself and the application only accesses the wrapper for sending a request or for receiving a response from the TPM.

Based on the functions available in the TPM, two methods for securing the communication between two nodes through Modbus TCP are presented. Based on the duration associated to the necessary operations, the HMAC-SHA256 is faster and more flexible from implementation point of view than ECDSA. Worth mentioning is that the most time-expensive operations during normal operation associated to the ECDSA are the ones related to the signing of the message with the generated private key and the validation of the signature using the public key. Nevertheless, the private cryptographic key used for ECDSA is created through TPM and stored securely in the device’s memory while the HMAC key is only loaded encrypted in the TPM, which brings an advantage from the security point of view for ECDSA. Both methods were subjected to several MITM attacks, but without success, proving the efficiency of the cryptographic implemented schemes against this type of attack.

As presented in the paper, hardware trusted modules are designed specifically for integration with industrial automation systems, in order to allow the addition of hardware security in the context of critical infrastructures and automation systems, which makes the presented proof of concept a subject of actuality with a real potential for being adopted in the industry. Additionally, the solution offers the possibility to reduce the interference on functioning legacy systems and to benefit from their initially foreseen lifecycle. 

## Figures and Tables

**Table 1:** Measurements of the executed trusted platform module (TPM) functions for primary key generation.

TPM Function	Duration (s)
TPM2_GetKeyTemplate_RSA_EK	0.000007
TPM2_CreatePrimaryKey	0.040547

**Table 2:** Measurements of the executed TPM functions done in a secure environment by a third party.

TPM Function	Duration (s)	Applicable to Proposed Method
TPM2_CreateAndLoadRSAKey	0.096907	HMAC-SHA256
TPM2_LoadExternal (RsaPublicKey)	0.002226	HMAC-SHA256
TPM2_RsaEncrypt	0.008255	HMAC-SHA256
TPM2_CreateAndLoadEccKey	0.122234	ECDSA ECC-256
TPM2_LoadExternal (EccPublicKey)	0.005993	ECDSA ECC-256

**Table 3:** Measurements of the executed TPM functions related to the authentication messages based on keyed-hash functions (HMAC) based proposed concept.

TPM Function	Duration (s)
TPM2_RsaDecrypt	0.159672
TPM2_LoadHashedKey	0.068886
TPM2_HmacStart	0.006950
TPM2_HmacUpdate	0.002146
TPM2_HmacFinish	0.008667

**Table 4:** Measurements of the executed TPM functions related to the ECDSA based proposed concept.

TPM Function	Duration (s)
TPM2_LoadEccPublicKey	0.005892
TPM2_Sign	0.068411
TPM2_VerifySignature	0.103083

**Table 5:** Analysis of the security strength and additional size for message *m.*

Proposed Method	Key Size (bit)	Key Strength (bit)	Output Size (bit)
HMAC-SHA256	256	256	256
ECDSA ECC-256	256	128	512
